# Confirmatory Analysis of Per and Polyfluoroalkyl Substances in Milk and Infant Formula Using UHPLC–MS/MS

**DOI:** 10.3390/molecules26123664

**Published:** 2021-06-16

**Authors:** Ovokeroye A. Abafe, Linda R. Macheka, Joshua O. Olowoyo

**Affiliations:** 1 Residue Analysis Laboratory, Agricultural Research Council-OVR, Pretoria 0110, South Africa; misslindamacheka@gmail.com; 2 School of Health Sciences, University of KwaZulu-Natal, Private Bag x5400, Durban 4001, South Africa; 3 School of Science and Technology, Sefako Makgatho Health Sciences University, Pretoria 0204, South Africa; joshua.olowoyo@smu.ac.za

**Keywords:** QuEChERS, confirmatory, UHPLC–MS/MS, breastmilk, retail milk, infant formula, liquid–liquid extraction

## Abstract

An ultra-high performance liquid chromatography tandem mass spectrometry method was developed and validated for the sensitive determination and unambiguous confirmation of residues of per and polyfluorinated alkyl substances (PFAS) in breastmilk, retail milk and infant formulas following two sample preparation methods. Sample pre-treatment was carried out by a simplified QuEChERS method without requiring dSPE or any further clean-up. The method was validated in accordance with the requirements of Commission Decision 657/2002/EC with slight modifications. The method displayed good linearity with R^2^ ranging from 0.9843–0.9998 for all target PFAS. The recovery and within-laboratory reproducibility of the method (*n* = 63) were in the range 60–121% and 5–28%, respectively. The decision limit, detection capability and limit of quantitation ranged from 30–60 ng kg^−1^ to 40–100 ng kg^−1^ and 5–50 ng kg^−1^, respectively. Acceptable matrix effect values in the range −45–29% were obtained with uncertainty of measurement lower than 25% for all target PFAS. The method displays its suitability for the sensitive and high-throughput confirmatory analysis of C_4_–C_14_ PFAS in breastmilk, dairy milk and infant formulas.

## 1. Introduction

Per and polyfluorinated alkyl substances (PFAS) belong to a broad group of chemicals comprising the perfluorocarboxylic acids (PFCAs) and perfluorosulfonic acids (PFSAs), characterized by a fully fluorinated hydrophobic carbon chain bonded to diverse hydrophilic heads [1].

The unique properties of these chemicals, such as chemical and thermal stability and their ability to lower surface tension, make them popular and very useful in various applications in food packaging materials, fire-fighting foams, fat and water repellents for paper, leather and textile treatment, performance chemicals and in the production of fluorinated polymers, among other applications [2,3].

The extreme environmental persistence, toxic and bioaccumulative properties of PFAS have resulted in concerns over their environmental fate and prevalence, hence several studies have established different pathways of human exposure to PFAS. These include the ingestion of house dust [4,5,6], consumption of food and water [3,7,8,9,10,11,12,13], and air inhalation [5,6], among others, with diet being the major exposure pathway for non-occupationally exposed populations [8,10,11,12].

The concentrations of PFAS in a broad category of food have been reported worldwide, however, only one report is currently available on PFAS in the South African domestic food supply [13]. Several studies have reported milk [13,14,15] and other dairy related products [16,17] as major sources of dietary exposure to PFAS due to the high yearly per capita consumption of milk across the globe, and also its use in numerous products such as prepared foods and baby formula, among others [11,18].

Liquid chromatography with tandem mass spectrometry (LC–MS/MS) is the technique of choice for the analysis of PFAS, as it offers the advantages of high selectivity and provides (pseudo) molecular ion and sufficient product ions utilized for the unambiguous confirmation of individual PFAS, and offers the great sensitivity needed for low-level detection of PFAS [14,15,16,17,19]. Dairy milk is a complex matrix for the analysis of persistent organic pollutants such as PFAS, owing to its composition of proteins, sugars and lipids [20,21]. Since PFAS accumulate in both protein and fat [15], careful sample preparation techniques need to be employed for accurate identification and quantitation. Several rigorous protein precipitation steps, lipid hydrolysis and alkaline digestion of milk have been employed as sample pre-treatment techniques to minimize matrix interferences [17,22,23]. Nevertheless, these have proven to be tedious, unsafe and expensive and sometimes not necessary, as they usually require large quantities of toxic organic solvents and often lead to losses in analyte recoveries [24,25,26].

Solid phase extraction (SPE) using mixed mode sorbent cartridges with several organic solvents, such as acetonitrile and methanol in the presence of formic acid, is popular in the literature for the analysis of PFAS in milk owing to the advantage of combining extraction and clean-up in a single step [8,9,10]. However, evidence suggests that formic acid could be a source of perfluorooctanoic acid (PFOA) impurities in PFAS analysis [8], hence the need for additional purification of solvents resulting in lengthy sample preparation time. Similarly, the use of SPE for clean-up has been documented to affect the recovery of PFAS from milk samples [15,27], with the majority of cartridges favoring the recovery of certain PFAS. For instance, the hydrophilic-lipophilic-balanced (HLB) sorbents offer good recovery only for long-chain PFAS, while traditional silica-based sorbents such as C18 are considered best for only PFOA and perfluorooctanesulfonic acid (PFOS) [11].

Although ion-pairing extraction (IPE) has been a good alternative, it is often followed by multiple clean-up steps, making the process resource and time consuming, hence unsuitable in high-throughput laboratories. Similarly, both IPE and SPE are often prone to the matrix effect due to co-extraction of other matrix components, and thus affect the final analysis of extracts [17]. Recently, Sadia et al. [20] employed both IPE and SPE methods following either acid or alkaline digestion for the analysis of three PFAS in various food matrices. The authors concluded that neither acid nor alkaline digestion was necessary for the analysis of PFAS in milk as no significant differences in PFAS concentrations were obtained between digestion with acid, alkaline or without any digestion.

The quick, easy, cheap, effective, rugged and safe (QuEChERS) method is well known to be accurate and highly productive for the ultra-trace determination of pesticides, veterinary drugs and other organic compounds in food matrices [24]. The use of this method for the analysis of PFAS in milk and biological samples is yet to be fully demonstrated, however, recent studies [24,25,26,28] have applied QuEChERS with multiple clean-up stages including the use of dispersive solid extraction (dSPE) and other sorbents, e.g., C18 for the analysis of PFAS. Recently, we reported the application of QuEChERS for the direct analysis of PFAS in dairy milk and infant formula [13], however, this method needs to be fully validated and extended to other matrices.

In the present study, we present a fully validated sensitive, selective, rapid, robust and cost effective ultrahigh-performance liquid chromatography tandem mass spectrometry (UHPLC–MS/MS) method for the simultaneous detection and quantification of eleven perfluorocarboxylic acids and four perfluorosulfonic acids in three matrices including breastmilk, dairy milk and infant formula using isotope labeled internal standards. The effectiveness of simplified QuEChERS and liquid–liquid extraction methods over conventional SPE using various cartridges were investigated for the analysis of PFAS. The validated method was applied for the analysis of PFAS in breastmilk, retail dairy milk and infant formula.

## 2. Results

### 2.1. Identification and Confirmation

The following identification and confirmation criteria were met in order for a target PFAS in a sample to be considered positive in accordance with the requirements of Commission Decision 2002/657/EC [29]. The ion ratio of the intensity of the two transitions (quantifier and qualifier MRMs) in a sample matched those obtained from the mean ion ratio of the matrix-matched calibration curve and was within the maximum permissible tolerance limit (i.e., for ion ratio >0.5, the maximum permitted tolerance is ±20%). The retention time of a given analyte in a sample must be within ±2.5% of the relative retention time of the same analyte in the matrix-matched calibration curve. The presence of a signal at each of the two MRMs for the target analyte in the sample was achieved, i.e., the use of one precursor ion and two product ions to achieve four identification points, fulfilling the Commission Decision 2002/657/EC on identification point requirements for compounds without established maximum residue limits.

### 2.2. Method Validation

#### 2.2.1. Selectivity

**Figure 1:**
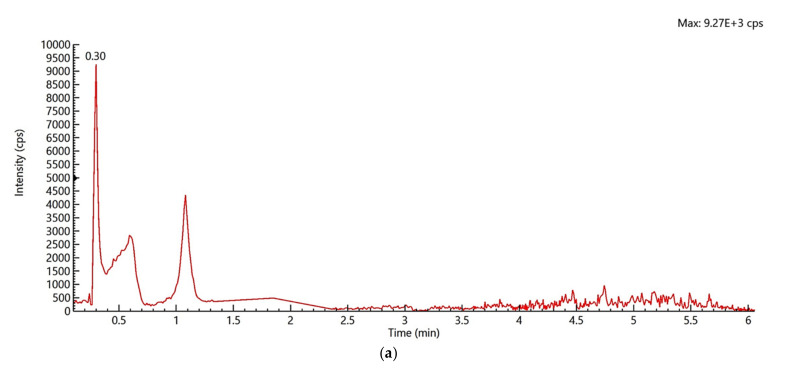

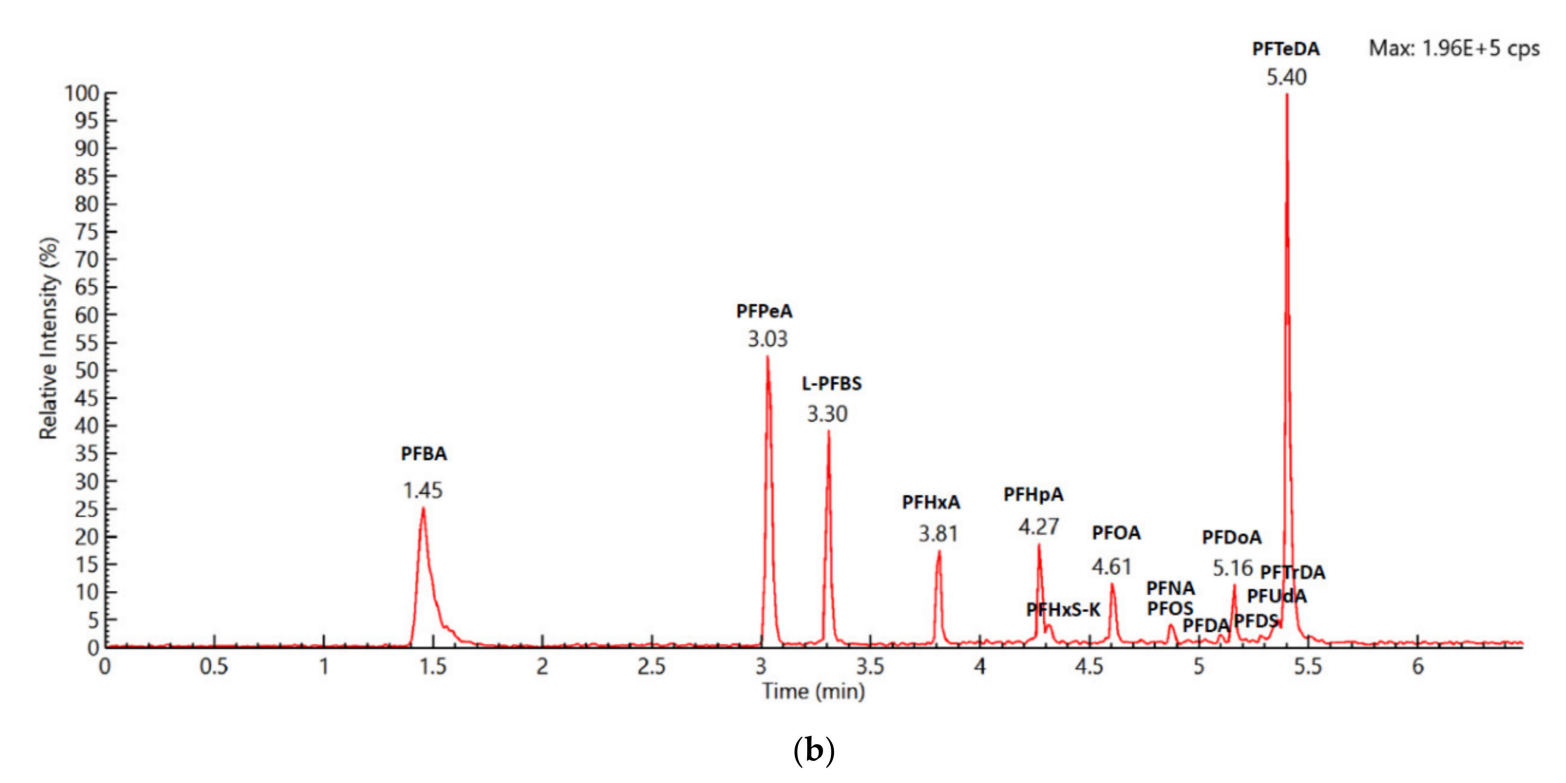
Selectivity of per and polyfluorinated alkyl substances (PFAS) in milk: (**a**) TIC of milk matrix blank; (**b**) TIC of spiked milk.

The selectivity of the method was evaluated by the analysis of 21 blank replicates for each matrix (milk and infant formula). The absence of any peak at the retention time of the target analyte indicated the absence of matrix interference that may lead to false positive signals (Figure 1a,b).

#### 2.2.2. Linearity

Good sensitivity was obtained for all target compounds, enabling the possibility of injecting a cocktail of all fifteen target PFAS in a matrix over the working range of the method. The ratio of the peak area of the quantitative ion pair of each standard to internal standard (Y) versus the concentration (x) in the range 5–1200 ng kg^−1^ was used for the determination of the analytical response for all target PFAS (Table 1).

#### 2.2.3. Recovery

Mean recoveries of each target analyte at the three validation levels were calculated using results from 21 replicates for each analyte (Table 2). Recoveries for each compound were determined by using the regression equations of the matrix-matched calibration curves for each analyte. The advantage of this parameter is that it indicates the recovery of each target analyte within the working range.

#### 2.2.4. Precision (Repeatability and within-Laboratory Reproducibility) and between-Laboratory Reproducibility

The precision (Table 2) of the method shown in terms of repeatability (intra-day repeatability) and within-laboratory reproducibility (inter-day repeatability) was expressed as the % RSD values of a set of 21 replicates each at the three validation levels (5, 50 and 100 ng kg^−1^). The reproducibility experiment lasted for three days.

To further elucidate the performance of the developed procedure, the method was applied for the determination of PFAS residues in NIST SRM 1954. Since there are no certified values for PFAS in SRM 1954, the mean concentration of PFAS in SRM 1954 reported using this method was compared with concentrations reported by other authors [30,31,32] in Table 3.

#### 2.2.5. Matrix Effect

The analyses of PFAS by MS/MS are often prone to matrix effects (ME) characterized by either signal enhancement or suppression, usually associated with the influence of co-eluting compounds in sample extracts. In this study, ME (Table 1) was evaluated following the method reported by González-Antuña et al. [33] as the ratio of the slope of the matrix-matched calibration curve and the slope of the calibration curve of standard solution in pure solvent at the same spiking range (Equation (1)):

(1) $$\mathrm{ME}\;\left(\%\right)=\left(1-\frac{{\mathrm{Slope}}_{\mathrm{matrix}\;\mathrm{matched}}}{{\;\mathrm{Slope}}_{\mathrm{solvent}}}\right)\times 100$$

#### 2.2.6. Decision Limit, Detection Capability and Limit of Quantitation

For the determination of the decision limit (CCα) and detection capability (CCβ); sixty-three matrix blank samples were fortified at the three validation levels (5, 50 and 100 ng kg^−1^). The CCα and CCβ (Table 1) were evaluated following Equations (2) and (3), in line with Commission Decision 657/2002/EC for substances without an established maximum residue limit (MRL):

(2) $$\mathrm{CC}\alpha =\frac{\left(\left(y_{a}+2.33\sigma_{N}\right)-y_{a}\right))}{b}$$

(3) $$\mathrm{CC}\beta =\frac{\left((y_{a}+2.33\sigma_{N}+1.64\;\sigma_{N})-y_{a})\right)}{b}$$

where $y_{a}\;$is the intercept of the calibration curve generated for the three validation levels; $\sigma_{N}$ is the standard deviation of $y_{a}$ and b is the slope of the calibration curve obtained for the three validation levels. An α and β-error of 1 and 5%, respectively was employed for each target PFAS.

The limit of quantitation (LOQ) of the method was determined for each analyte as the smallest concentration in the calibration curve for each analyte with a signal to noise ratio greater than 10.

#### 2.2.7. Robustness

**Figure 2:**
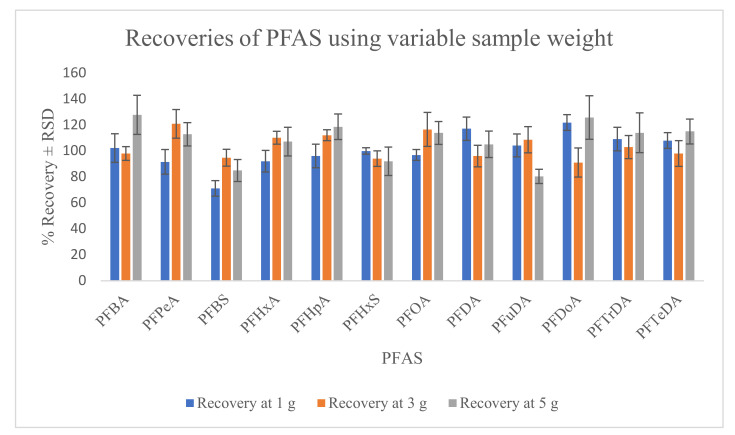
Recovery of PFAS using varying sample weight.

The robustness of the method was examined by varying different parameters including the weight of sample utilized for extraction (1, 3 and 5 g), variation in particle size of two different chromatographic columns and variation in the concentration of mobile phase buffer solution used. The obtained data showed that varying sample weight does not have significant (*p*-value > 0.05) influence on the recovery of PFAS (Figure 2), similar to the observation obtained for the variation of the concentration (5–15 mM) of buffer used in the mobile phase. However, varying the particle size of the chromatographic column had significant influence on peak shape and chromatographic separations (Supplementary Materials Figure S1a–c).

#### 2.2.8. Measurement Uncertainty

The Nordtest approach of a single laboratory validation was used for the estimation of measurement uncertainty in line with our previous study [34] with slight modifications. The combined standard uncertainty (*Uc*) is made up of the intermediate precision *u*(Rw) obtained from the pooled standard deviation of routine spiked quality control samples, and the uncertainty due to laboratory bias, u(bias), obtained from uncertainty due to the method bias acquired from the recoveries of inter-analyst reproducibility of spiked samples (Equation (4)):

(4) $$Uc=\sqrt{u{\left(\mathrm{Rw}\right)}^{2}+u{\left(\mathrm{bias}\right)}^{2}}$$

The *Uc* of each PFAS determined at 95% confidence level ranged from 9.7–24.2% (Table 1). This is deemed acceptable considering the low analytical range in the sub-nanogram concentration range [29]. This is the first study estimating an uncertainty budget for PFAS in milk.

#### 2.2.9. Stability

Although it is well noted that PFOS and PFOA are persistent organic pollutants, it is important to evaluate the stability of PFAS in various matrices and under various conditions. The stability of PFAS was determined at two concentrations of 100 ng kg^−1^ and 1500 ng kg^−1^ spiked in matrix (*n* = 3) and prepared in solvent (*n* = 3) and kept at 4 °C over a 90-day period. The % stability (*ST*) was calculated using the initial peak area (*S*_0_) obtained on day zero and the peak area determined after the introduction of pauses with duration (*S_t_*); i.e., peak area obtained for the same matrix extract and solvent standards on day 30, day 60 and day 90, as shown in Equation (5).

(5) $$ST\;\left(\%\right)=\frac{S_{t}}{S_{0}}\times 100\%$$

## 3. Discussion

### 3.1. Method Development

The most crucial aspect in the analysis of PFAS in milk is the sample pre-treatment method. The binding of PFAS to proteins, particularly albumins, has led many investigators to employ protein precipitation using methanol, acetonitrile or freeze-drying [8,9,10,11] and hydrolysis using protease and lipase enzymes to break down milk emulsion, thereby releasing PFAS bound in fat and proteins in order to improve detection and quantitation. In this study, we evaluated the effectiveness of three different extraction techniques: liquid–liquid extraction; solid phase extraction (evaluating four different cartridges with various sorbent packing–Oasis^®^ HLB, Sep-Pak^®^ C_18_, Oasis^®^ MCX from Waters Corporation, Milford, MA, USA and Varian Bond Elut^®^ from Agilent Technologies, Santa Clara, CA, USA) as described in Supplementary Materials Sections S1 and S2; and a simplified QuEChERS procedure (described in Section 4.2.3). The result obtained from the evaluation of the SPE cartridges showed that Varian Bond Elut^®^ C_18_ and Oasis^®^ MCX inefficiently recovered C_6_–C_14_ PFAS, and Sep-Pak^®^ C_18_ and Oasis^®^ HLB cartridges recovered almost all the target PFAS with recoveries ranging from 30–130% and 30–109%, respectively. The combination of hydrophilic–lipophilic sorbent in the Oasis HLB cartridge allows for the extraction of acidic, neutral and basic compounds at neutral pH, accounting for the high capacity, good and reproducible recoveries for most target PFAS with the HLB cartridge, hence it was selected for further evaluation in comparison with the liquid–liquid and simplified QuEChERS extractions.

**Figure 3:**
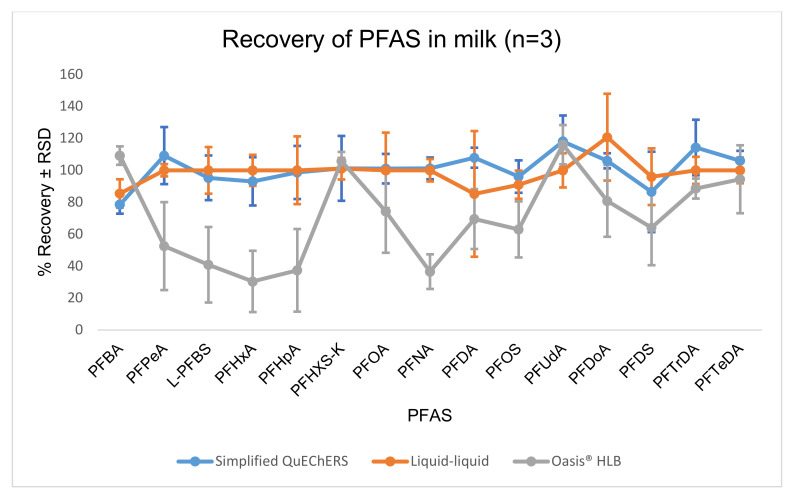
Recovery (*n* = 3) of PFAS in milk using the simplified QuEChERS, liquid–liquid and Oasis HLB SPE cartridge.

The recovery and precision of PFAS identification using the three extractions methods are presented in Figure 3. As observed, comparable data were obtained for both liquid–liquid and the simplified QuEChERS extracts for most of the target PFAS. No statistically significant difference (*p* = 0.5113) was observed for the results obtained for liquid–liquid and the simplified QuEChERS extracts. The obtained results were not surprising as acetonitrile, employed as the extraction solvent for both liquid–liquid and simplified QuEChERS extractions, is well known to be effective in the precipitation of protein and fat, thereby releasing bound PFAS in milk [35]. Though comparable results were obtained, the blockage of the electrospray ionization source was observed after multiple injections (>200 injections) of the liquid–liquid extracts. This observation could be associated with the aggregation of small molecules and precipitation of residual fat and protein, hence the simplified QuEChERS procedure was optimized, fully validated and applied in this study.

Several chromatographic parameters, such as column temperature, injection volume, flow-rate and concentrations of organic modifier were optimized. Methanol provided the best separation for all PFAS. Two volatile organic modifiers (ammonium formate and ammonium acetate) at different concentrations were optimized for the separation of PFAS. We found 10 mM of ammonium acetate suitable for the complete resolution of all target analytes with improved sensitivity, though concentrations ranging from 1–5 mM of ammonium acetate as mobile phase modifier are mostly reported in the literature for the chromatographic separation of PFAS [3,4,36]. The improved chromatographic peak shape, selectivity and higher signal to noise ratio obtained is attributed to the well-known advantages of high pH mobile phases in negative electrospray ionization MS [37].

Chromatographic separation of PFAS was achieved first by evaluating three different chromatographic columns: Kinetex^®^ C_18_ (1.7 µm: 2.1 × 100 mm), Kinetex^®^ Biphenyl (2.5 µm: 2.1 × 50 mm), Phenomenex^®^, Torrance, CA, USA and PerkinElmer^®^ Brownlee Superficially Porous Particles (SPP) C_18_ (2.7 µm: 2.1 × 100 mm), Perkin Elmer, Inc., Waltham, MA, USA. While poor chromatographic separation, especially for the perfluorosulfonic acids, was observed for the biphenyl column, the two C_18_ columns efficiently separated all target PFAS (Figure S1a–c). Although the 2.7 µm SPP C_18_ column gave higher chromatographic peak response with minimum co-eluting analytes, the 1.7 µm Kinetex^®^ column gave better chromatographic peak shape owing to its smaller particle size, hence the Kinetex column was selected for use. Optimized injection volume of 10 μL and pump flow rate of 0.800 mL min^−1^ gave optimum performance for all target analytes in this study (Figure S2).

**Figure 4:**
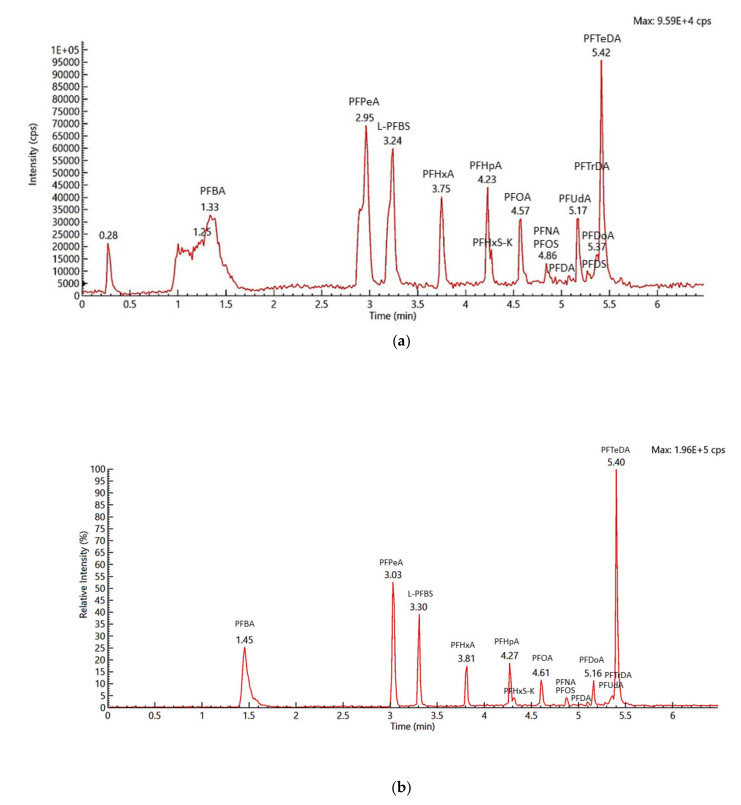
(**a**) Total ion chromatogram of 10,000 ng kg^−1^ PFAS spiked in milk in MRM acquisition mode. (**b**) Improved total ion chromatogram of 10,000 ng kg^−1^ PFAS spiked in milk in time-managed MRM acquisition mode.

**Figure 5:**
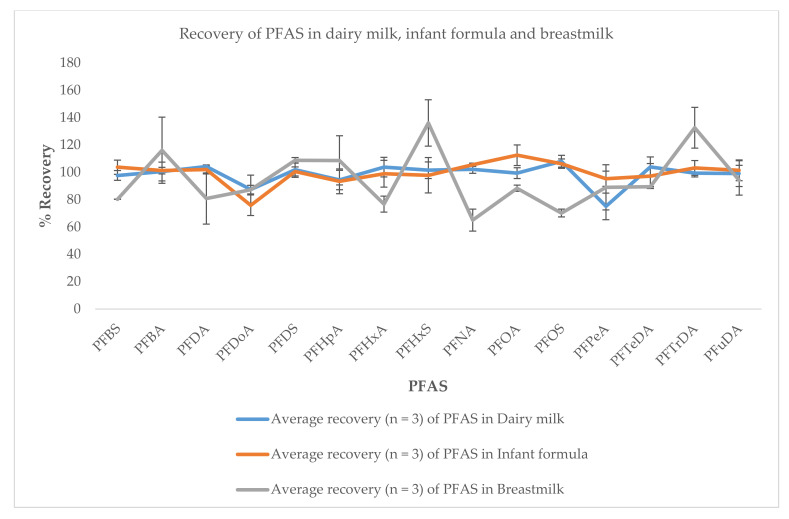
Recovery of PFAS in dairy milk, infant formula and breastmilk.

In the MS/MS method, at least two MRM transitions corresponding to the molecular ion or pseudomolecular ion together with two product ions (obtained through direct MS/MS infusion of native standards of individual PFAS) were used to unequivocally confirm and quantify the presence and amount of PFAS. The most intense MRM was selected as the quantitative MRM and the second and third (where available) MRMs were utilized as qualitative MRMs. Sensitivity of the method was improved by automatically generating the MS acquisition method by defining the expected retention time of analytes and their corresponding time window in the time-managed MRM acquisition, hence allowing for sufficient dwell time for monitoring multi-analytes in samples. This parameter improved both the resolution and sensitivity of PFAS, as shown in Figure 4a,b, thus allowing for very low-level determination of PFAS in milk. A single factor analysis of variance (α = 0.05) showed that there was no significant difference (*p*-value = 0.7074) between the recoveries of PFAS in milk and infant formula, thus indicating the suitability of the method for the determination of PFAS in these matrices (Figure 5).

### 3.2. Method Validation

The validated method satisfied all performance characteristics evaluated in this study, thereby displaying the suitability of the method for the multi-residue determination of PFAS in milk and infant formula. Acceptable linear regressions were obtained for all target compounds over the concentration range. The determination coefficient (R^2^) values ranged from 0.9843–0.9998 (see Table 1) for all target PFAS in the matrix-matched calibration curves. These results showed acceptable linearity in the chosen working range. The recoveries of PFAS ranged from 65–136%. These values were generally within acceptable limits for all target analytes (i.e., ±40% for mass fractions lesser than or equal to 1 µg kg^−1^), indicating the suitability of the method for the multi-residue analysis of PFAS. The extraction method and instrumental determination worked very well for all target PFAS in this study, with recoveries generally better than recoveries reported in the literature for similar matrices [9,10,11,12,14,15]. The within-day repeatability (*n* = 7) of measurement was generally <15% for all target analytes. The within-laboratory reproducibility (*n* = 21) for each spiking level was mostly lower than 30% except for PFDoA with % RSD of 34 at the lowest spiking level of 5 ng kg^−1^ (Table 2). The mean concentrations of PFAS reported in SRM 1954 using this method were consistent with those reported by Awad et al. 2020, Keller et al. 2010 and Nyberg et al. 2018 [30,31,32] for the majority of PFAS (Table 3). The results display satisfactory performance and indicate the suitability of the method for routine analysis of this class of compounds in breastmilk, dairy milk and infant formula.

The obtained ME for all PFAS ranged from −20–13% (Table 1) with the exception of PFBS (29%) and PFDoA (−45%), which displayed obvious signal enhancement and suppression, respectively. ME values less than 50% are deemed acceptable. The ME value is indicative of the suitability of the validated sample preparation and instrumental methods proposed in this study. The noticeable matrix effect obtained for PFBS and PFDoA could be associated with the fact that only two radiolabeled compounds were employed as internal standards for the quantitation of all target PFAS, hence the use of a corresponding labeled internal standard for each PFAS may improve the quantitation of PFDoA and PFBS. The ME values obtained are truly reflective of the entire working range in this study, unlike most studies of PFAS where ME are evaluated using single concentration levels [36].

The decision limit (CCα) and detection capability (CCβ) of PFAS determined in this study ranged from 30–50 ng kg^−1^ and 40–100 ng kg^−1^, respectively (Table 1). These values indicate the ability of the method to unequivocally determine these PFAS, for which no maximum residue limits are established for milk and infant formula. To our knowledge, this is the first study estimating CCα and CCβ for PFAS in milk. The values obtained for all PFAS showed the possibility of the application of the method for regulatory testing of a wide range of short and long chain PFAS in milk. The LOQ of the method was determined for each analyte as the lowest concentration levels in the calibration curve with a signal to noise ratio greater than 10. The obtained LOQs for the target analytes varied from 5–50 ng kg^−1^ (Table 1). Generally, these limits of quantitation are considered acceptable and mostly better than LOQs reported in the literature for similar matrices [10,11,20,21,26,35], considering that we practically demonstrated the LoQ values reported in this study, unlike the theoretical LOQ values mostly reported in literature. This displays the possibility of the application of the method to low-level regulatory detection of PFAS in milk and infant formulas [13].

As expected, the majority of target PFAS (Supplementary Materials Table S2) were stable in the matrix over a 90-day period stored at 4 ± 2 °C, with the exception of PFBA, which demonstrated reduced recovery of 40% and 55% after 60 days and 90 days storage, respectively; low recovery (33%) of PFBA in solvent was observed after 90 days of storage. This is similar to the ~50% recovery observed for PFPeA starting from 60 through 90 days of storage. The obtained stability data show that the majority of PFAS in milk extracts and pure solvent could be stored for a period of 90 days at 4 °C without noticeable loss in integrity.

### 3.3. Application to Real Sample

The validated method was applied for the determination of fifteen PFAS in thirteen breastmilk samples obtained from nursing mothers, and eight pooled dairy milk and two pooled infant formulas obtained from local supermarkets, in addition to the application of this method in our recently published study [13]. At least three PFAS (Table S3) were detected in all the samples with concentrations ranging from <LOQ–317 ng L^−1^ to <LOQ–259 ng kg^−1^ and <LOQ–294 ng kg^−1^ for breastmilk, infant formulae and dairy milk, respectively. While PFDA, PFuDA, PFDoA and PFTrDA were most prevalent in retail milk, PFBA, PFPeA, PFBS and PFHxA were more prevalent in infant formula and breastmilk. The results show an overall dominance of perfluorocarboxylic acids compared to perfluoroalkylsulfonic acids in both milk and infant formulas, with PFBS being the only perfluoroalkyl sulfonate detected (57 ng kg^−1^) in the infant formulae samples.

## 4. Materials and Methods

### 4.1. Chemicals

Ammonium acetate and sodium dihydrogen orthophosphate monohydrate were obtained from Sigma Aldrich, Johannesburg, South Africa. Merck (Pty) Ltd., Johannesburg, South Africa supplied liquid chromatography grade (99.9%) acetonitrile and methanol. High purity deionized water was obtained from an Elgastat UHQ water purifier system with resistivity of 18.2 Ωm. SPE cartridges—Waters Oasis^®^ HLB 6cc (200 mg), Waters Sep-Pak^®^ C18 3cc (500 mg) and Waters Oasis^®^ MCX 3cc (60 mg) were obtained from Microsep (Pty) Ltd., Johannesburg, South Africa, and Varian Bond Elut^®^ 3 mm (500 mg) was obtained from Chemetrix (Pty) Ltd., Midrand, South Africa. QuEChERS extraction pouches containing 4 g magnesium sulfate and 1 g sodium chloride were products of Agilent Technologies obtained from Chemetrix (Pty) Ltd., Midrand, South Africa. High-purity perfluorocarboxylic acid standards including perfluorobutanoic acid (PFBA), perfluoropentanoic acid (PFPeA), perfluorohexanoic acid (PFHxA), perfluoroheptanoic acid (PFHpA), perfluorooctanoic acid (PFOA), perfluorononanoic acid (PFNA), perfluorodecanoic acid (PFDA), perfluoroundecanoic acid (PFUdA), perfluorododecanoic acid (PFDoA), pefluorotridecanoic acid (PFTrDA) and perfluorotetradecanoic acid (PFTeDA) and the four perfluorosulfonic acid standards including linear perflurorobutanesulfonic acid (PFBS), perfluorohexanesulfonic acid (PFHxS), perfluorooctanesulfonic acid (PFOS) and perfluorodecanesulfonic (PFDS) were purchased from Wellington Laboratories (Guelph, ON, Canada). The carbon labeled internal standards (^13^C3-PFOA and ^13^C3-PFNA) were purchased from Cambridge Isotope Laboratories, Inc. (Tewksbury, MA, USA). The purity of all the analytical standards ranged from 97–99.9%. Standard Reference Material (SRM) 1954 (Organic Contaminants in Fortified Human Milk) was obtained from the National Institute of Standard and Technology (NIST), Gaithersburg, MD, USA.

### 4.2. Standard and Sample Preparation

#### 4.2.1. Preparation of Standard Solution

A 1.0 mg L^−1^ stock solution containing a cocktail of all fifteen PFAS was prepared in methanol. From this, working standard solutions of 10 µg L^−1^ and 1000 ng L^−1^ were prepared in methanol. Stock solution was kept at −20 ± 5 °C while working solutions were kept at 4 ± 2 °C.

#### 4.2.2. Sampling

The milk and infant formulas were purchased from registered retail stores in Pretoria, South Africa. Samples were stored at 4 ± 2 °C throughout the analysis.

#### 4.2.3. Simplified QuEChERS Method

One gram of sample was weighed into a 50 mL centrifuge tube. The samples were spiked with 2 ng mL^−1^ of ^13^C PFNA and ^13^C PFOA as internal standards and vortexed for 30 s. This was followed by adding 10 mL each of acetonitrile and ultrapure water. QuEChERS extraction packs containing 4g MgSO_4_ and 1 g NaCl were added and dissolved in the mixture by vigorous agitation. The mixture was vortexed for 10 s and then centrifuged at 8000 rpm for 10 min, set at 4 °C. Approximately 500 μL of the supernatant was collected and filtered through a 0.22 μm syringe filter into an autosampler vial for UHPLC–MS/MS analysis. The results obtained following this protocol were compared with results obtained following liquid–liquid and solid phase extraction as detailed in Supplementary Materials Sections S1 and S2.

### 4.3. Instrumental Method

The chromatographic separation was achieved with a Perkin Elmer LX-50 UHPLC system (Perkin Elmer,(Perkin Elmer, MA, USA, MA, USA) equipped with a Kinetex^®^ C18 1.7 µm: 2.1 × 100 mm column, Phenomenex^®^, Torrance, CA, USA. The column oven temperature was kept at 50 °C. Chromatographic separation was achieved with a gradient consisting of 10 mM ammonium acetate in water (A) and methanol (B), at a constant flow rate of 0.8 mL min^−1^. An initial mobile phase flow of 90% A was held for 5.0 min and then decreased to 20% A for 4.0 min. This was followed by column wash with 100% B for 3.0 min and then followed by equilibration with the initial gradient composition of 90% A for 3.7 min. The total run time was 15.7 min. The injection volume was 10 μL.

Mass spectrometric identification and confirmation of PFCAs and PFSAs was achieved using a PerkinElmer^®^ QSight™ 220 triple quadruple mass spectrometer (MS/MS) operated in the negative electrospray ionization mode, with an electrospray voltage set at –4000 V. Nitrogen was used as drying and nebulizer gas, set at 140 and 400, respectively. The optimized hot surface-induced desolvation (HSID) temperature was set to 320 °C while the ion source temperature was set at 350 °C. The acquisition of PFAS was achieved using the time-managed multiple reaction monitoring (MRM) mode. At least two MRM transitions corresponding to a minimum of four identification points were monitored for each target analyte (Table S1). The collision energies (CE), entrance voltages (EV) and cell exit potential (CCL2) were individually optimized for each analyte through a flow injection analysis of individual PFAS standard solutions (Table S1). Data were acquired by using Perkin Elmer Simplicity™ 3Q software (version 1.4.1806.29651).

### 4.4. Method Validation

The developed method was validated for selectivity, accuracy, precision (in terms of repeatability and within-laboratory reproducibility), matrix effect, decision limit, detection capability, limit of quantitation, robustness, measurement uncertainty and stability in accordance with the requirements of Commission Decision 657/2002/EC with slight modifications as described in Section 2.2.

### 4.5. Quality Control

The experimental work described in this study was carried out in an ISO 17025 accredited laboratory employing strict quality control and quality assurance measures: retail milk and infant formula samples which previously tested negative for the target PFAS were used for preparation of matrix blanks used for the validation experiments and calibration curves. Since the use of the corresponding ^13^C3-labeled internal standard for each of the 15 target PFAS was not feasible, two labeled PFAS—^13^C3-PFOA and ^13^C3-PFNA were utilized. The choice of IS used for the quantification of individual PFAS was based on the relative recovery and relative standard deviation (*n* = 21) obtained for each PFAS. For each batch of analysis, a check standard was analyzed at the beginning and the end of each batch to monitor fluctuation in chromatographic separations. Spiked quality control samples (*n* = 3) were analyzed for each batch of analysis to monitor the method’s performance. Similarly, solvent blanks were analyzed after each sample run to monitor possible carryover effect. Method blanks (*n* = 6) treated as an unknown sample (without the use of an actual sample) were analyzed for each batch of analysis to monitor possible contamination through the analysis. None of the target PFAS was detected above limits of quantitation (LOQ) in the matrix blank. The performance of the developed method was further validated by the analysis of SRM 1954 (Organic Contaminants in Fortified Human Milk).

### 4.6. Statistics

Descriptive statistics such as mean, median, standard deviations and parametric tests such as the analysis of variance (ANOVA) were carried out by using Microsoft^®^ Excel 2016, Microsoft Corporation, Albuquerque, NM, USA. Non-parametric statistical analysis was carried using XLSTAT 2019, Addinsoft, Paris, France. All values lower than LOQ were treated as zero.

## 5. Conclusions

In the present study, a simple, sensitive, robust and very cost effective confirmatory method using simplified QuEChERS for the multi-residue determination of fifteen PFAS in three matrices of breastmilk, dairy milk and infant formula by UHPLC–MS/MS was developed. The method was extensively validated with fortified milk and infant formula and excellently satisfied all performance criteria in accordance with the requirements of Commission Decision 657/2002/EC. The CCα and CCβ were sufficiently low for the food safety determination of residues of PFAS in dairy milk and infant formulas as well as breastmilk. The method fulfils all performance characteristics, thereby displaying its fitness for purpose in the confirmatory analysis of PFAS in dairy milk, infant formula and breastmilk.

## Figures and Tables

**Table 1:** Analytical validation parameters for PFAS in matrices.

Analyte	Internal Standard	CCα/ng kg^−1^	CCβ/ng kg^−1^	LOQ/ng kg^−1^	Regression Equation	R^2^	% ME	MU
L-PFBS	^13^C-PFOA	30	50	5	Y = 3.2844x − 0.0041	0.9993	29	20.2
PFBA	^13^C-PFOA	30	60	50	Y = 1.2464x + 0.1871	0.9983	−20	19
PFDA	^13^C-PFNA	30	50	5	Y = 10.604x + 1.2112	0.9963	−15	12.6
PFDoA	^13^C-PFNA	50	90	5	Y = 7.5313x + 2.3109	0.9957	−45	26
PFDS	^13^C-PFNA	40	60	5	Y = 6.9454x + 0.0345	0.9919	−19	14.5
PFHpA	^13^C-PFOA	50	80	5	Y = 3.0588x + 0.3459	0.9981	−3	13.4
PFHxA	^13^C-PFOA	30	60	5	Y = 6.4299x + 0.0239	0.9989	6	12.3
PFHxS	^13^C-PFOA	30	50	50	Y = 5.184x + 0.2849	0.9998	13	12.7
PFNA	^13^C-PFNA	30	50	5	Y = 10.478x + 0.0657	0.9941	5	10.5
PFOA	^13^C-PFOA	30	50	5	Y = 9.3557x+ 0.1439	0.9997	−11	12.3
PFOS	^13^C-PFNA	30	50	5	Y = 8.2593x + 0.4787	0.9843	−20	11.9
PFPeA	^13^C-PFOA	60	100	5	Y = 3.0567x + 0.3481	0.9979	−6	24.2
PFTeDA	^13^C-PFNA	30	40	5	Y = 8.7075x + 1.549	0.9989	−20	9.7
PFTrDA	^13^C-PFNA	30	50	5	Y = 10.627x + 3.0888	0.9959	−17,5	12.2
PFuDA	^13^C-PFNA	40	60	5	Y = 11.602x + 2.5914	0.9905	−17	15.3

ME: matrix effect; MU: measurement uncertainty.

**Table 2:** Accuracy and precision of PFAS tests.

Analyte	Spiked Concentration (ng kg^−1^)	Within-Laboratory Repeatability (*n* = 7)		Within-Laboratory Reproducibility (*n* = 21)	
		**Recovery (%)**	**Precision (%)**	**Recovery (%)**	**Precision (%)**
L-PFBS	5	100	7.6	83	28
	50	100	4	93	14
	100	100	7.1	100	11
*PFBA					
	50	104	9.6	98	14
	100	100	13	100	18
PFDA	5	100	15.9	100	16
	50	100	10.8	100	11
	100	105	5.4	100	8.6
PFDoA	5	120	18.2	100	34
	50	120	11.5	110	22
	100	100	6.8	95	18
PFDS	5	100	10.9	122	22
	50	100	11.5	110	14
	100	100	7.1	105	6
PFHpA	5	100	11.3	100	13
	50	100	6.4	100	12
	100	100	14.2	95	16
PFHxA	5	100	5.7	100	11
	50	110	4.3	100	16
	100	105	8.6	100	13
*PFHxS					
	50	100	7.8	90	13
	100	95	7.1	97	7
PFNA	5	100	9.6	100	10
	50	100	9	100	13
	100	100	7.6	100	7
PFOA	5	100	10.3	100	17
	50	100	8.4	106	10
	100	100	7.2	106	9
PFOS	5	100	5.5	112	15
	50	110	11.7	107	10
	100	101	6.2	113	8
PFPeA	5	100	18	60	25
	50	105	14.9	100	21
	100	100	10.3	105	20
PFTeDA	5	100	8.4	100	15
	50	100	4.6	100	5
	100	100	6.9	100	7
PFTrDA	5	100	14.4	100	17
	50	90	11.5	93	12
	100	100	7.4	100	6
PFuDA	5	100	5.3	100	25
	50	100	5.8	94	12
	100	95	6.5	104	20

* The LOQ of PFBA and PFHxS were 50 ng kg^−1^, hence only two validation levels were quantified.

**Table 3:** Comparison of mean PFAS concentration (ng mL^−1^ ± SD) in NIST SRM 1954 measured in this study with those concentrations reported by other authors.

PFAS	This Study	Keller et al., 2010 [30]	Nyberg et al., 2018 [31]	Awad et al., 2020 [32]
PFBA	<LOQ	NR	NR	NR
PFPeA	0.0262 ± 0.0159	NR	0.0158 ± 0.023	NR
PFBS	0.0802 ± 0.0003	0.007	0.0034 ± 0.0044	0.004 ± 0.0017
PFHxA	0.0068 ± 0.0012	0.014 − 0.023	0.07 ± 0.052	NR
PFHpA	0.010 ± 0.0007	0.014 ± 0.001	0.0125 ± 0.007	0.011 ± 0.010
PFHxS	0.0156 ± 0.0169	0.012 − 0.031	0.0177 ± 0.0046	0.0198 ± 0.006
PFOA	0.0211 ± 0.012395	0.116 − 0.810	0.074 ± 0.003	0.093 ± 0.038
PFNA	0.0498 ± 0.0026	0.016 − 0.104	0.0157 ± 0.003	0.016 ± 0.0051
PFOS	0.0573 ± 0.045	0.136 − 0.189	0.097 ± 0.0017	0.0934 ± 0.031
PFDA	0.022 ± 0.0078	0.006 − 0.127	0.0083 ± 0.0043	0.0092 ± 0.0044
PFUdA	0.00815 ± 0.0031	0.007 − 0.094	0.0034 ± 0.001	0.0069 ± 0.0039
PFDS	0.0908 ± 0.022	NR	0.0009 ± 0.0008	0.002 ± 0.0023
PFDoA	0.0027 ± 0.00280	0.003 − 0.044	0.0013 ± 0.0005	0.0026 ± 0.0018
PFTrDA	0.0254 ± 0.0023	0.199	0.001 ± 0.001	0.0036 ± 0.0035
PFTeDA	0.0045 ± 0.0000317	0.002	0.0017 ± 0.0015	0.0048 ± 0.00

NR: Not reported.

## Data Availability

The data presented in this study are available in the Supplementary Materials section.

## Supplementary Materials

Figure S1a–c: (a) Chromatographic Separation of PFASs using Kinetex® Biphenyl (2.5 µm, 2.1 × 50 mm) column. (b) Chromatographic Separation of PFASs using Kinetex® C18 (1.7 µm, 2.1 × 100 mm) column. (c) Chromatographic Separation of PFASs using Brownlee Superficially Porous Particles (SPP) C18 (2.7 µm: 2.1 × 100 mm) column; Table S1: Optimised MS/MS parameters for the analysis of PFASs; Scheme S1: Representative Fragmentation pattern of PFOA; Table S2: Stability of PFAS in matrix and solvent; Table S3: Concentrations of PFAS in infant formula, retail milk and breastmilk; 1: Liquid–liquid extraction procedure; S2. Solid phase extraction procedure: Figure S2a–c. Chromatographic separation of PFAS at variable injection volumes (a) 10 µL (b) 20 µL and (c) 30 µL.

## Sample Availability

Samples of the compounds are not available from the authors.
