# Supplementary material for: Confirmatory Analysis of Per and Polyfluoroalkyl Substances in Milk and Infant Formula Using UHPLC–MS/MS
Source: Molecules. 2021 Jun 16;26(12):3664. doi: 10.3390/molecules26123664 (PMC8234569; doi:10.3390/molecules26123664)
Supplement: Supplementary file 1 [file molecules-26-03664-s001.zip › molecules-1165958-supplementary.pdf]

Supplementary Material

Confirmatory Analysis of Per and Polyfluoroalkyl Substances in Milk and Infant Formula Using UHPLC–MS/MS

Ovokeroye A. Abafe [1,2,*], Linda R. Macheka [1,3] and Joshua O. Olowoyo [3]

[1] Agricultural Research Council-OVR, Residue Analysis Laboratory, Pretoria 0110, South Africa; misslindamacheka@gmail.com
[2] School of Health Sciences, University of KwaZulu-Natal, Private Bag x5400, Durban 4001, South Africa
[3] School of Science and Technology, Sefako Makgatho Health Sciences University, Pretoria 0204, South Africa; Joshua.Olowoyo@smu.ac.za;
* Correspondence: abafeo@arc.agric.za or chiralpops@gmail.com

S1. Liquid-liquid extraction procedure

One gram of milk sample was weighed into 50 mL polypropylene tube. The samples were spiked at three validation levels (5, 50 and 100 ng L^{-1}) using the cocktail working standard solution and 100 ng of a mixture of the two internal standards. The mixture was vortexed for 10 sec, followed by the addition of 20 mL acetonitrile and then vortexed for a further 30 sec and centrifuged at 7500 rpm for 10 min, set at 4 °C. The supernatant was concentrated under nitrogen to approximately 0.1 mL in a Turbovap set at 40 °C. The extract was reconstituted with 900 μL of the initial gradient of mobile phase and filtered through 0.22 μm syringe filter into an LC autosampler vial.

S2. Solid phase extraction procedure

The following SPE cartridges Oasis® HLB 6 cc (200 mg), Waters Sep-Pak® C_{18} 3cc (500 mg), Oasis® MCX 3 cc (60 mg) and Varian Bond Elut® 3 mm (500 mg) were evaluated for the recovery of PFAS from dairy milk. Approximately, 1 mL extracts obtained through the experiment described above were further cleaned-up with each of the SPE sorbents. The cartridges were activated and conditioned with 2 mL methanol, 2 mL water and 2 mL of 0.05 M phosphate buffer (pH 8.5). Samples were passed through the cartridge and washed with 3 mL of 0.05 M phosphate buffer (pH 8.5), followed by 1 mL of water. PFAS were eluted with 3 mL of acetonitrile at a flow rate of approximately 0.5 mL min^{-1}. The eluates were concentrated to incipient dryness under nitrogen to approximately 0.1 mL in a Turbovap set at 40 °C. The residue was reconstituted with 0.9 mL of initial gradient of mobile phase and filtered through a 0.22 μm syringe filter into an LC autosampler vial for UHPLC-MS/MS analysis.

Table S1. Optimised MS/MS parameters for the analysis of PFASs.

Compound Name	Acronym	Retention Time (minutes)	Precursor Ion (m/z)	Product Ion (m/z)	Fragmentation	CE	EV	CCL2
Perfluorobutanoic Acid	PFBA	1.45	212.9	69	$[M - C_2F_4COOH]^-$	80	−20	100
				169	$[M - COOH]^-$	14	−20	100
Perfluoropentanoic Acid	PFPeA	3.03	262.9	218.9	$[M - COOH]^-$	14	−20	100
				69	$[M - C_3F_6COOH]^-$	57	−20	100
Perfluorobutanesufonic Acid	PFBS	3.30	299.5	80	$[M - C_4F_9]^-$	80	−20	100
				99	$[M - C_4F_8]^-$	42	−20	100
Perfluorohexanoic Acid	PFHxA	3.81	313.1	119	$[M - C_3F_6COOH]^-$	30	−20	100
				269	$[M - COOH]^-$	14	−20	100
Perfluoroheptanoic Acid	PFHpA	4.27	363	318.9	$[M - COOH]^-$	18	−20	100
				169	$[M - C_3F_6COOH]^-$	28	−20	100
Perfluorohexanesulfonic Acid	PFHxS	4.31	399	80	$[M - C_6F_{13}]^-$	83	−20	100
				99	$[M - C_6F_{12}]^-$	44	−20	100
				119.1	$[M - C_4F_8SO_3]^-$	45	−20	100
Perfluoroctanoic Acid	PFOA	4.61	412.8	368.9	$[M - COOH]^-$	18	−20	100
				169	$[M - C_4F_8COOH]^-$	33	−20	100
Perfluorononanoic Acid	PFNA	4.78	462.8	169	$[M - C_5F_{10}COOH]^-$	30	−20	100
				419	$[M - COOH]^-$	19	−20	100
				219	$[M - C_4F_8COOH]^-$	22	−20	100
Perfluorooctanesulfonic Acid	PFOS	4.80	499.1	80	$[M - C_8F_{17}]^-$	85	−20	100
				99	$[M - C_8F_{16}]^-$	57	−20	100
Perfluorodecanoic Acid	PFDA	5.00	512.9	169	$[M - C_6F_{12}COOH]^-$	30	−20	100
				469	$[M - COOH]^-$	17	−20	100
				219	$[M - C_5F_{10}COOH]^-$	23	−20	100
Perfluoroundecanoic Acid	PFUdA	5.27	563.1	169	$[M - C_7F_{14}COOH]^-$	32	−20	110
				519	$[M - COOH]^-$	17	−20	100

				318.9	[M – C_4F_8COOH]$^-$	21	−20	100
Perfluorodecanesulfonic Acid	PFDS	5.21	599.1	80	[M – $C_{10}F_{21}$]$^-$	110	−20	100
				99	[M – $C_{10}F_{20}$]$^-$	57	−20	100
Perfluorododecanoinc Acid	PFDoA	5.16	612.8	169	[M – $C_8F_{16}COOH$]$^-$	42	−20	100
				569	[M – COOH]$^-$	18	−20	100
				318.9	[M – $C_5F_{10}COOH$]$^-$	26	−20	100
Perfluorotridecanoic Acid	PFTrDA	5.36	662.8	619	[M – COOH]$^-$	16	−20	120
				368.9	[M – $C_5F_{10}COOH$]$^-$	26	−20	120
				169	[M – $C_9F_{18}COOH$]$^-$	40	−20	140
Perfluorotetradecanoic Acid	PFTeDA	5.40	712.9	468.9	[M – C_4F_8COOH]$^-$	25	−20	100
				668.9	[M – COOH]$^-$	18	−20	100
				168.9	[M – $C_{10}F_{20}COOH$]$^-$	48	−20	100

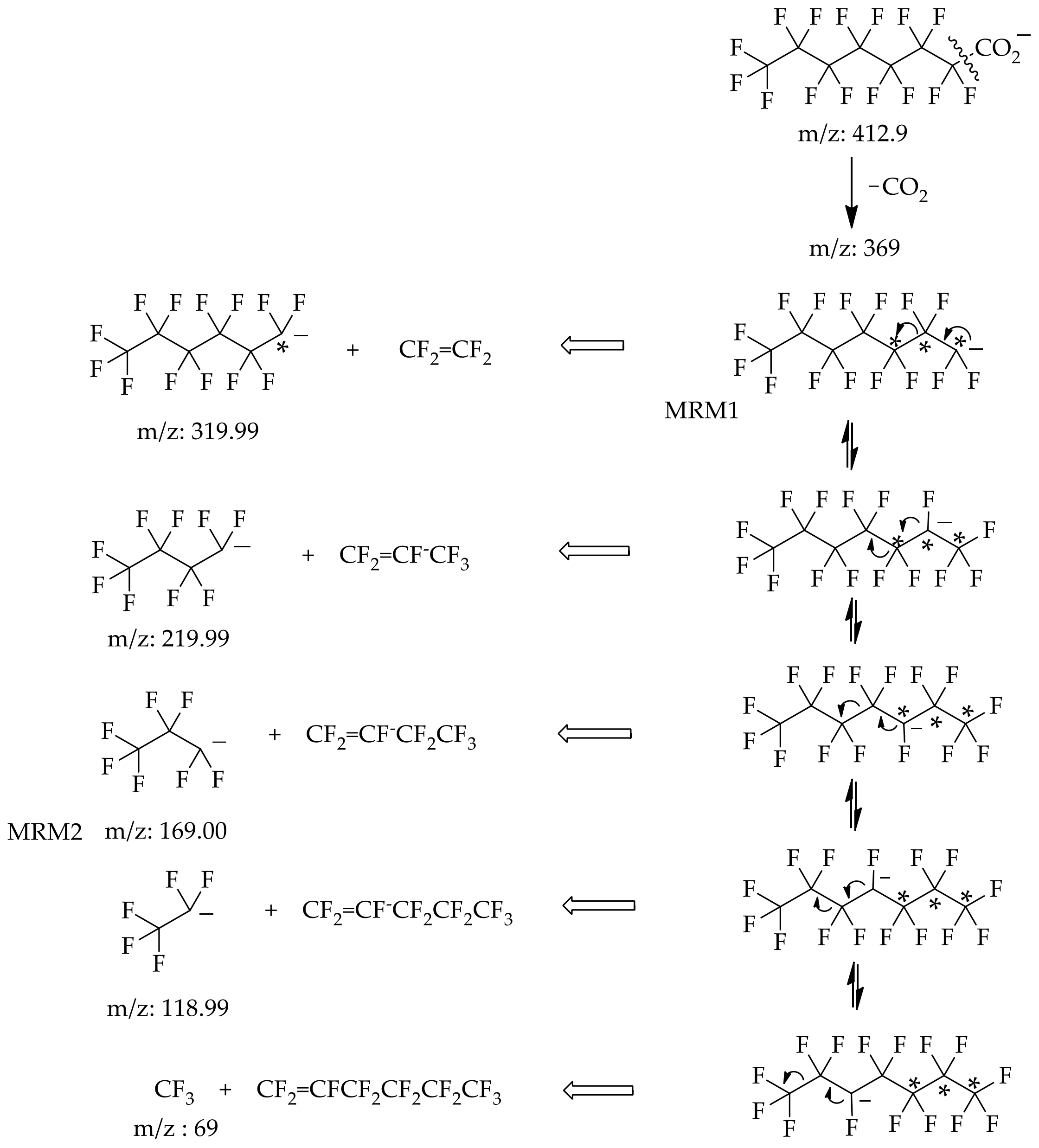

Scheme S1. Representative Fragmentation pattern of PFOA

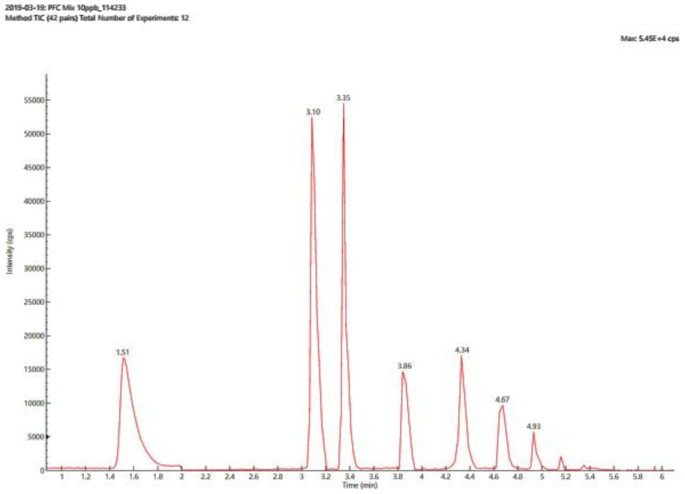

(**a**)

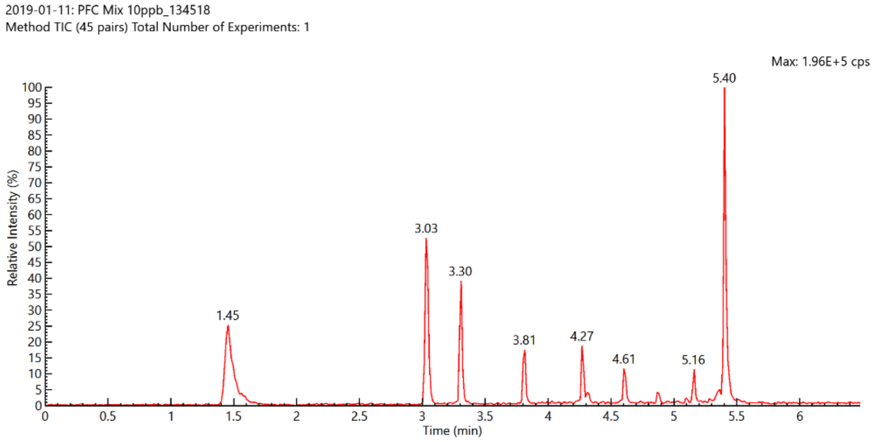

(**b**)

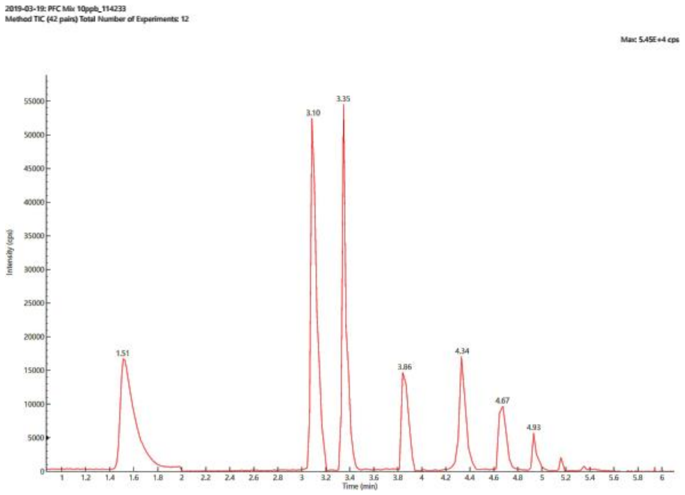

(**c**)

Figure S1. (**a**) Chromatographic Separation of PFASs using Kinetex® Biphenyl (2.5µm, 2.1 x 50 mm) column. (**b**) Chromatographic Separation of PFASs using Kinetex® C18 (1.7µm, 2.1 x 100 mm) column. (**c**) Chromatographic Separation of PFASs using Brownlee Superficially Porous Particles (SPP) C18 (2.7µm: 2.1 x 100 mm) column.

Table S2. Stability of PFAS in matrix and solvent.

Analyte	Month 1		Month 2		Month 3		Month 1		Month 2		Month 3	
	% Stability in matrix (100 ng kg^{-1})	% Stability in solvent (100 ng L^{-1})	% Stability in matrix (100 ng kg^{-1})	% Stability in solvent (100 ng L^{-1})	% Stability in matrix (100 ng kg^{-1})	% Stability in solvent (100 ng L^{-1})	% Stability in matrix (1500 ng kg^{-1})	% Stability in solvent (1500 ng L^{-1})	% Stability in matrix (1500 ng kg^{-1})	% Stability in solvent (1500 ng L^{-1})	% Stability in matrix (1500 ng kg^{-1})	% Stability in sol-vent(1500 ng L^{-1})
PFBA	101	103	60	138	66	119	107	114	58	114	45	67
PFPeA	100	108	99	66	104	86	100	114	101	50	106	53
L-PFBS	90	106	98	109	96	107	103	98	101	101	105	106
PFHxA	100	100	98	112	107	101	100	100	101	102	101	104
PFHpA	130	100	72	117	90	106	101	100	98	100	101	104
PFHxS	100	100	100	94	95	108	101	99	105	106	100	106
PFOA	100	100	94	96	101	117	102	156	100	158	101	162
PFNA	80	100	124	110	114	128	105	99	108	90	100	94
PFOS	100	70	145	89	106	94	95	116	109	95	100	118
PFDA	80	130	102	80	110	98	100	69	124	70	104	72
PFUDA	100	100	99	105	105	103	103	108	110	88	104	87
PFDS	100	130	112	124	91	111	105	84	124	79	104	83
PFDoA	113	118	127	98	105	98	102	91	125	94	107	96
PFTrDA	116	100	116	101	119	102	96	89	121	87	92	85
PFTeDA	113	106	100	105	97	100	97	74	125	67	101	80

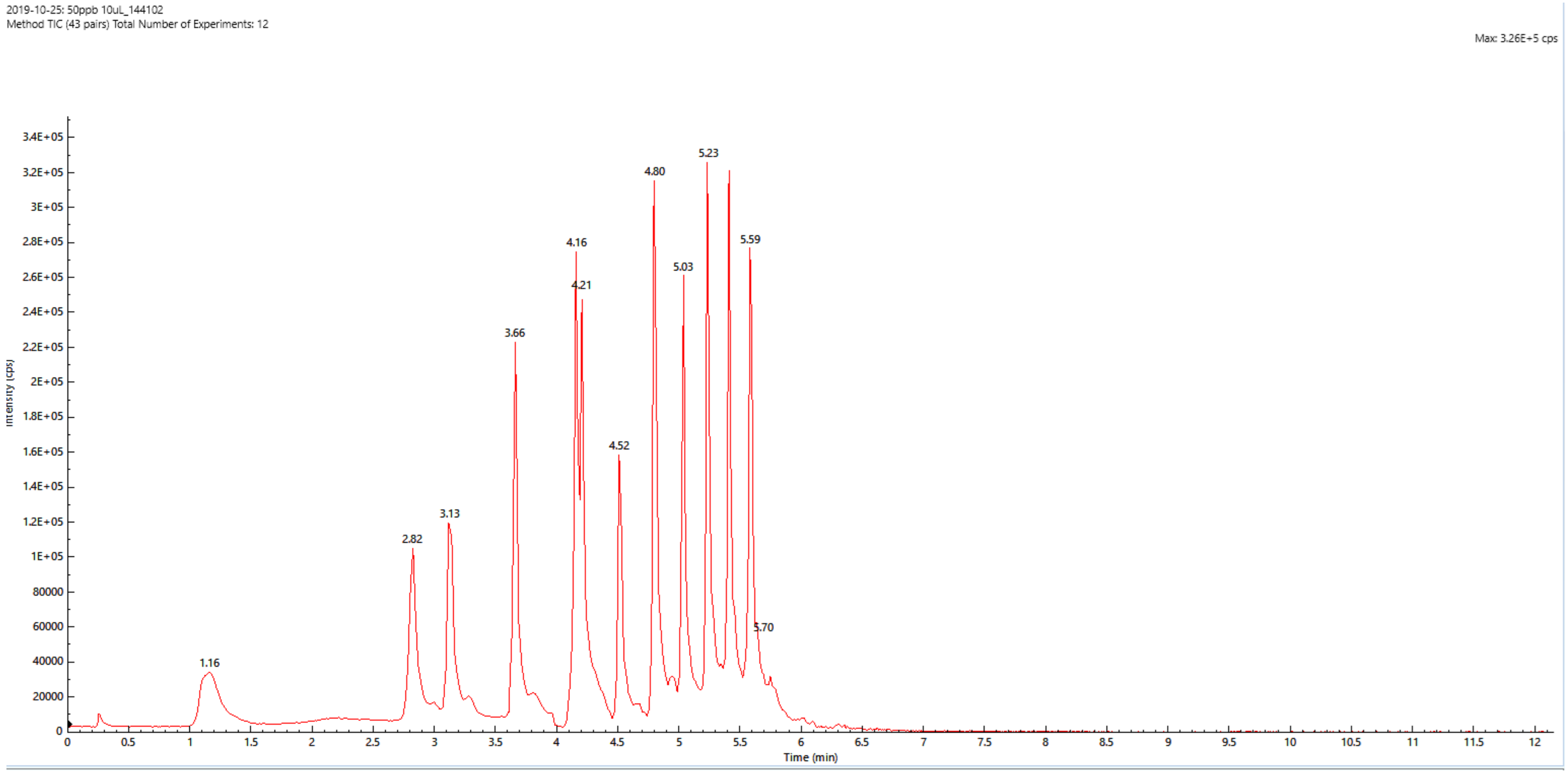
2019-10-25: 50ppb 10uL_144102
Method TIC (43 pairs) Total Number of Experiments: 12
Max: 3.26E+5 cps
Intensity (cps)
Time (min)
1.16
2.82
3.13
3.66
4.16
4.21
4.52
4.80
5.03
5.23
5.59
5.70

(a)

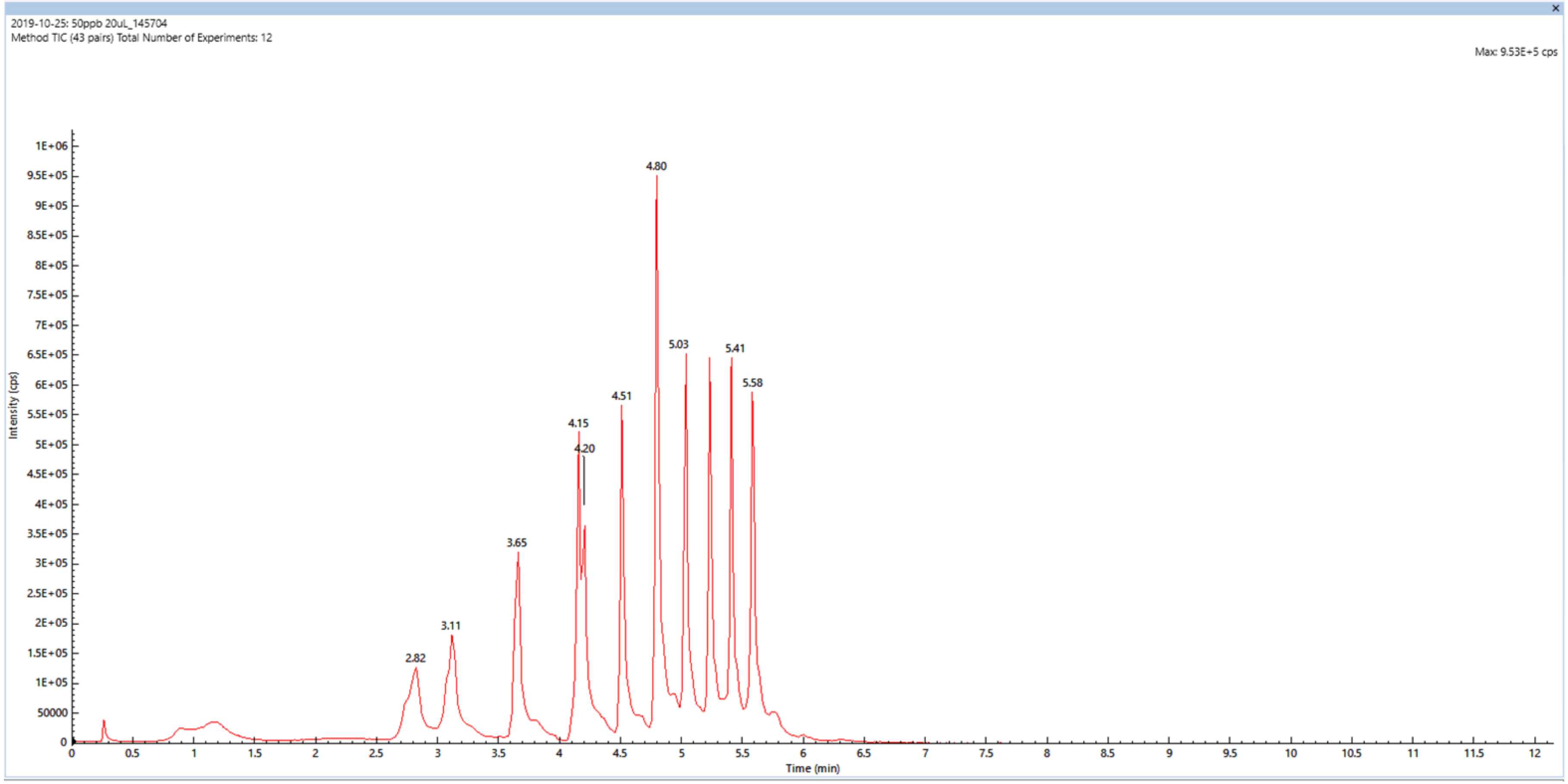
2019-10-25: 50ppb 20uL_145704
Method TIC (43 pairs) Total Number of Experiments: 12
Max: 9.53E+5 cps
Intensity (cps)
Time (min)
2.82
3.11
3.65
4.15
4.20
4.51
4.80
5.03
5.41
5.58

(b)

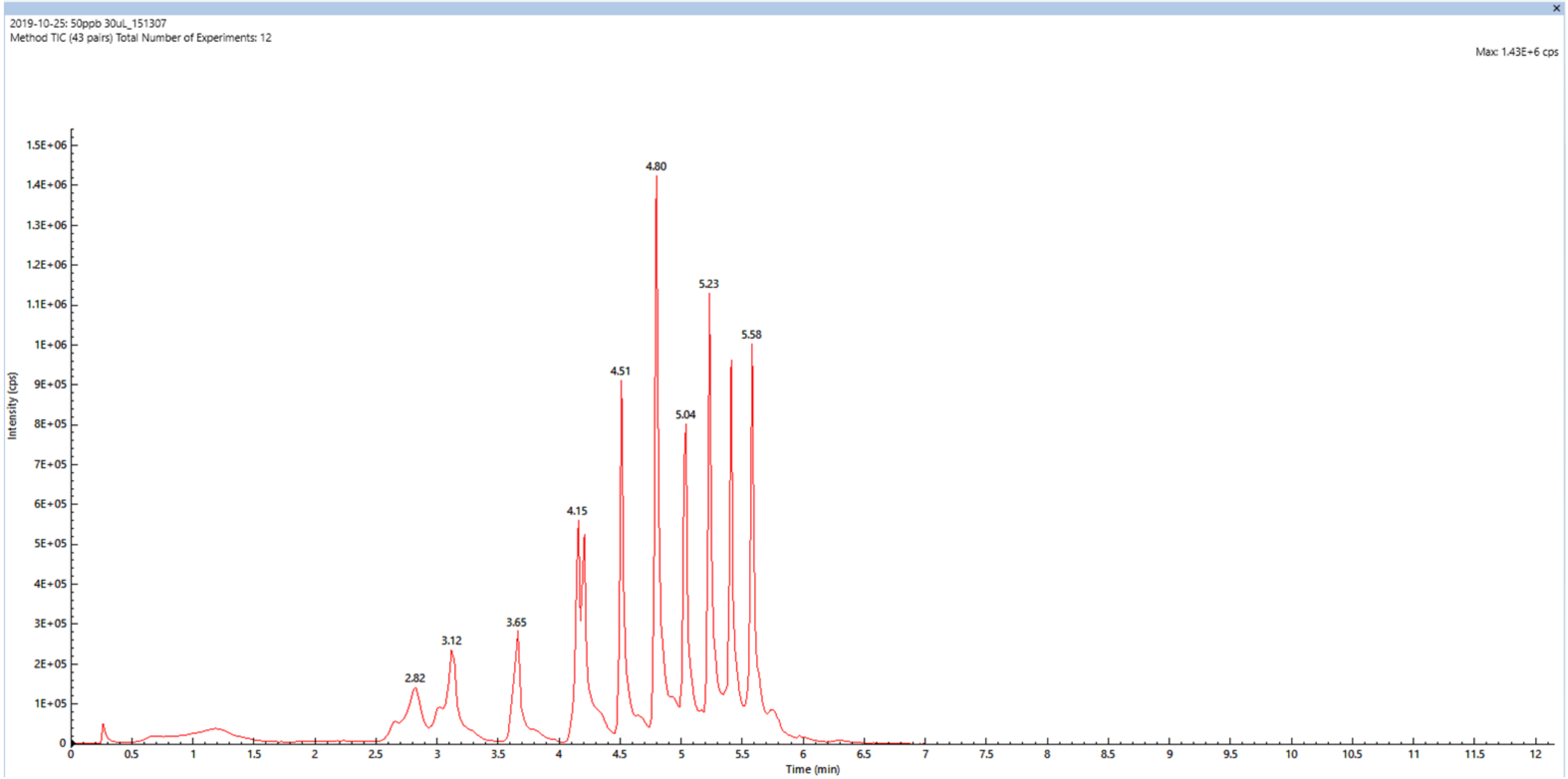

(**c**)

Figure S2. Chromatographic separation of PFAS at variable injection volumes (**a**) 10 µL (**b**) 20 µL and (**c**) 30 µL.

Table S3. Concentrations of PFAS in infant formula, retail milk and breastmilk.

Infant formula/ µg kg⁻¹	**PFBA**	**PFPeA**	**PFBS**	**PFHxA**	**PFHpA**	**PFHxS**	**PFOA**	**PFNA**	**PFOS**	**PFDA**	**PFUdA**	**PFDS**	**PFDoA**	**PFTrDA**	**PFTeDA**
Sample 1	0.017	0.045	0.005	0.010	0.021	< LOQ	0.022	< LOQ	< LOQ	< LOQ	< LOQ	< LOQ	0.027	0.115	< LOQ
Sample 2	0.259	0.069	0.01	0.013	0.037	< LOQ	< LOQ	< LOQ	< LOQ	0.054	< LOQ	< LOQ	0.097	0.106	< LOQ
Sum	0.276	0.114	0.015	0.023	0.058	< LOQ	0.022	< LOQ	< LOQ	0.054	< LOQ	< LOQ	0.124	0.221	< LOQ
Mean	0.138	0.057	0.0075	0.012	0.029	< LOQ	0.022	< LOQ	< LOQ	0.054	< LOQ	< LOQ	0.062	0.112	< LOQ
**Retail Milk / µg kg⁻¹**															
Sample 3	0.080	0.060	< LOQ	0.010	< LOQ	< LOQ	0.090	< LOQ	0.120	0.080	0.130	< LOQ	0.294	0.140	0.110
Sample 4	< LOQ	0.080	0.050	< LOQ	< LOQ	< LOQ	0.050	< LOQ	0.090	0.10	< LOQ	< LOQ	0.160	0.240	0.140
Sample 5	< LOQ	0.040	0.030	< LOQ	< LOQ	< LOQ	0.030	< LOQ	< LOQ	0.10	0.180	< LOQ	0.230	0.180	< LOQ
Sample 6	0.050	0.030	0.010	0.020	0.06	< LOQ	0.100	0.05	0.08	0.120	0.10	< LOQ	0.180	0.220	0.220
Sample 7	< LOQ	< LOQ	< LOQ	0.07	< LOQ	< LOQ	0.060	< LOQ	0.040	0.050	< LOQ	< LOQ	0.230	< LOQ	< LOQ
Sample 8	0.080	< LOQ	< LOQ	< LOQ	0.030	< LOQ	0.130	0.030	< LOQ	0.110	< LOQ	< LOQ	0.120	< LOQ	0.180
Sample 9	0.060	0.040	< LOQ	0.040	0.050	0.030	0.080	0.040	0.060	0.080	0.10	0.050	< LOQ	0.260	0.220
Sample 10	< LOQ	0.060	0.020	< LOQ	< LOQ	< LOQ	0.050	< LOQ	< LOQ	0.10	0.080	< LOQ	0.160	0.10	0.160
Sum	0.190	0.310	0.110	0.140	0.140	0.030	0.590	0.120	0.390	0.740	0.590	0.050	1.374	1.140	1.030
Mean	0.024	0.039	0.014	0.020	0.020	0.004	0.070	0.015	0.05	0.09	0.074	0.006	0.172	0.143	0.129

Breastmilk/ ng ml^{-1}

Sample 11	0.20	0.210	0.050	0.10	0.140	0.130	0.220	< LOQ	< LOQ	0.210	0.140	0.030	0.210	0.140	0.060
Sample 12	0.160	0.317	0.130	0.080	0.010	< LOQ	0.190	0.120	0.030	< LOQ	0.20	< LOQ	0.160	0.230	< LOQ
Sample 13	0.09	< LOQ	< LOQ	< LOQ	< LOQ	0.080	0.280	< LOQ	0.060	< LOQ	0.090	< LOQ	< LOQ	0.090	0.050
Sample 14	< LOQ	0.180	< LOQ	0.030	< LOQ	0.160	0.130	0.090	< LOQ	0.140	0.170	< LOQ	< LOQ	0.160	< LOQ
Sample 15	0.230	0.220	0.050	< LOQ	< LOQ	< LOQ	< LOQ	0.160	0.040	0.080	0.080	< LOQ	0.110	0.130	0.090
Sample 16	0.140	0.190	0.060	< LOQ	< LOQ	0.040	0.260	0.080	0.090	0.10	0.060	< LOQ	0.080	< LOQ	< LOQ
Sample 17	< LOQ	0.260	< LOQ	0.10	< LOQ	0.130	0.160	< LOQ	< LOQ	< LOQ	< LOQ	0.150	< LOQ	0.080	< LOQ
Sample 18	< LOQ	0.220	< LOQ	< LOQ	< LOQ`	0.060	0.240	< LOQ	0.050	< LOQ	0.110	< LOQ	< LOQ	0.140	0.050
Sample 19	0.160	0.140	0.080	< LOQ	0.030	< LOQ	< LOQ	< LOQ	< LOQ	0.240	0.180	< LOQ	0.050	0.090	0.050
Sample 20	0.240	0.120	< LOQ	0.050	< LOQ	< LOQ	0.120	0.140	< LOQ	< LOQ	< LOQ	< LOQ	< LOQ	0.190	< LOQ
Sample 21	< LOQ	0.190	< LOQ	< LOQ	0.070	< LOQ	0.270	0.080	< LOQ	< LOQ	0.240	0.180	< LOQ	< LOQ	< LOQ
Sample 22	< LOQ	0.280	< LOQ	0.080	< LOQ	0.130	0.120	< LOQ	0.090	0.050	< LOQ	0.130	< LOQ	0.140	0.110
Sample 23	< LOQ	0.10	< LOQ	< LOQ	< LOQ	< LOQ	< LOQ	0.110	< LOQ	< LOQ	0.050	< LOQ	0.110	< LOQ	< LOQ
Sum	1.220	2.427	0.370	0.440	0.250	0.730	1.990	0.780	0.360	0.820	1.271	0.490	0.720	1.390	0.410
Mean	0.094	0.187	0.028	0.034	0.019	0.056	0.153	0.06	0.028	0.063	0.10	0.038	0.055	0.107	0.032
